# Avoidance Distance in Sheltered Cows and Its Association with Other Welfare Parameters

**DOI:** 10.3390/ani9070396

**Published:** 2019-06-28

**Authors:** Arvind Sharma, Clive J. C. Phillips

**Affiliations:** Centre for Animal Welfare and Ethics, School of Veterinary Science, The University of Queensland, Gatton Campus, Gatton 4343, Australia

**Keywords:** human–animal relationship, cow shelters, avoidance distance, welfare, assessment

## Abstract

**Simple Summary:**

Avoidance distance is an indicator of the welfare of farmed animals, which is routinely included in contemporary animal welfare assessments. Determination of this parameter for cows in traditional Indian shelters is important because the shelters owe their existence due to the concept of non-violence and compassion to animals, which is held by the Hindu majority population in India. These cows have outlived their economic value due to old age, reproductive problems, and/or subsequent abandonment by their owners. Their slaughter is prohibited by law in most Indian states. The avoidance distance test measures the closest distance that a person can approach a cow, which is a measure of whether stockpersons handling the cows in shelters have had a humane and positive relationship with these cows. Association of the avoidance distance was observed with the cows’ health, in particular their dirtiness and extent of musculoskeletal, digestive, and respiratory problems. This study provides information to justify monitoring this critical—but often neglected—aspect of cow shelter management for a sustainable improvement in sheltered cow welfare.

**Abstract:**

The human–animal relationship is an important welfare parameter in animal welfare assessment in cows, and the avoidance distance of cows to a stranger at the feed bunk is measured to assess this relationship. The assessment of the human–animal relationship in cow shelters in India, where old, unproductive, and abandoned cows are sheltered, is important to explore the welfare of cows in these shelters. The cows observed were of indigenous Indian breeds and breeds which were crosses between indigenous breeds and pure bred exotic cows. The human–animal contact in this context is of particular interest for welfare assessment as traditional Indian farming and sheltering systems involves regular close human–animal contact. In a cross-sectional study across 6 states, 54 cow shelters were visited and 30 cows in each shelter were randomly selected (1620 in total) for the assessment of avoidance distance and other cow-based (27 parameters) and resource-based (15 parameters) welfare parameters. Avoidance distance was assessed 1 h after morning feeding. Cows standing at the feeding manger were approached from the front at a rate of one step/s, starting 2 m away from the manger. The distance between the assessor’s hand and the cow’s head was estimated at the moment the cow moved away and turned its head, using a four-point scale (0, touched; 1, 0–50 cm; 2, 51–100 cm; and 3, >100 cm). The majority, 52%, of the cows allowed touch by the assessor and another 32% allowed approach within 50 cm, demonstrating tolerance, or even solicitation of close human–animal relationships by the cows. Avoidance distance increased with the proportion of cows with dirty hind limbs, tarsal joint swellings, and hair loss, and the extent of rumen fill. There was also evidence of reduced avoidance distances in cows with high levels of body condition score (BCS), dirty flanks, tarsal joint ulceration, carpal joint injuries, diarrhoea, hampered respiration, lesions on the body due to traumatic injuries, and body coat condition, probably as a result of moving difficulties. The avoidance distance was thus related to the health and welfare of the cows, providing a vital insight into the factors affecting human–animal contact in the shelters.

## 1. Introduction

Fear of people can be major source of stress in animals resulting in physiological changes in animals and negative effects on animal welfare [1]. The human–animal relationship, defined as the mutual perception of human and animal manifested in their mutual behaviour [2], is an important parameter in any dairy cow welfare assessment protocol. The quality of stockpersonship affects the welfare of animals in the performance of routine tasks such as feeding, cleaning, etc. [3]. Aversive handling of cows reduces their milk productivity [4,5,6]. This could be due to restlessness and nervous activity, stress hormone effects on lactogenesis or cows withholding their milk in the parlour as response to a stressful situation. Assessment of this relationship underlines the importance of stockpersonship in animal welfare [7]. Negative behaviour and handling of animals induces stress and risks injury to animals as well as humans [8].

Measurement of avoidance behaviour is important in assessment of animal behaviour because it demonstrates initial responses of an animal towards a change in the human environment [9]. Pioneering work on this aspect of animal behaviour as an indicator of poor welfare was initiated in experiments on pigs, in which they were found to be highly fearful of humans, with a pronounced stress response [10,11]. Subsequently, studies on avoidance behaviour as a response to fear of humans were initiated in sheep and cattle [12,13]. Measurement of avoidance distance (AD) of cows at a feed bunk to an approaching human is now an established test of the human–animal relationship. However, the results are dependent on several factors, including the animal’s genetic predisposition, the situation in which the test is conducted, and previous interactions of the animals with humans [14,15]. Avoidance distance (AD) has recently been included as an important welfare indicator in most contemporary cattle welfare assessments protocols in different parts of the world [16,17,18,19,20].

Animal husbandry in India usually involves close contact of humans with animals due to the traditional non-mechanized animal production operations practiced in many parts of the country. India has the largest cattle population in the world [21], and cow slaughter is not permitted by law in most of its states [22,23]. The surplus, old, abandoned, and non-productive cows are sheltered in age-old traditional shelters known as ‘gaushalas’ until they die due to natural causes. The points of contact between the cows and the stockpersons in cow shelters are substantially different than the conventional farming because these cows have no economic value. Most of the shelters have indoor housing and hence the cows are more dependent on human care than in farms. Close contact of cows with humans is normal as a result of the strong socio-cultural functions of the cows in the Indian context. Therefore, the assessment of human–animal relationships becomes more important in order to investigate whether cows are treated well in the shelters. In shelters, AD is likely influenced by the extent of habituation of the cows with people [24]. Regular contact with humans who deliver feed to the cows at the feed bunks may result in reduced AD at the feed bunks, but this may not be generalized to other situations. Nevertheless, a simple visual contact, particularly of a person providing food without any negative experiences, has a positive effect on the human–animal relationship [2]. Research on AD in dairy cattle suggests that it is not ‘context-specific’, i.e., the behaviour of cows under a variety of different type of AD tests is significantly related to the AD at the feed bunk test [7,25]. Human–animal relationships are a dynamic process, and changes in human behaviour towards animals can improve this relationship [2]. Fear of humans in cattle can be reduced within 2–5 weeks through routine positive behaviour [26,27]. However, cows learn to differentiate between positive and negative interaction between two different individuals, and their previous experience with handlers at a place affects their avoidance distance towards their handlers as well as a stranger [4,28].

Most of the studies on human–animal relationships have emphasized the role of stockmanship on productivity rather than the welfare of animals. As well as the factors described above, it is highly likely that AD will be affected by cow health, but there is a paucity of literature on this [29]. Disease which impairs movement may reduce AD, but other diseases may be related to a negative perception of humans, who may have treated them badly or been involved in their treatment for the disease with the involvement of pain and distress. The objective of our study was to assess the human–animal relationship in cow shelters through the measurement of AD at the feed bunk (manger) and explore the relationship with other cow disease and shelter-based welfare assessment parameters. It is part of a larger research project on the welfare assessment of cows in shelters in India, the results of which have been published previously [30,31]. To the best of our knowledge, no studies exist on the assessment of human–animal relationships on sheltered cows, for whom profitability is not the goal but perpetuation of cow welfare is the only motivation, mandated by religion and culture. There are isolated studies [32] on the relationship between AD and comprehensive cow health measures, which could be important in the incorporation into cow welfare assessments.

## 2. Materials and Methods

This study was conducted with animal and human ethics approval from the University of Queensland’s Animal and Human Ethics Committees (approval numbers SVS/CAWE/314/16/INDIA and 2016001243, respectively). A total of 54 cow shelters (gaushalas) located in six states of India (Gujarat, Maharashtra, Rajasthan, Punjab, Haryana, and Himachal Pradesh) were used for the study. These states either have large numbers of shelters and a traditional history of sheltering of cows, or newly established shelters. These six states are located in the north, west, and northwest India. A single two-day visit to each shelter occurred between December 2016 and July 2017. Out of the 54 shelters, 26 were selected by state veterinary officers and the Animal Welfare Board of India (AWBI), and the remaining shelters were obtained using a snowballing technique, taking recommendations from shelter managers. There was no significant difference (*p* < 0.05) in any measured parameter between shelters obtained by the two methods, when compared by analysis of variance or a Moods median test (in the case of non-normal residuals). In each shelter, 30 cows were randomly sampled, following a power calculation to determine the required numbers of cows and shelters [33], to detect an odds ratio of 4 with a power of 0.8 and α = 0.05. The sample size of 30 cows was sufficient to estimate within-herd prevalence with an error of 10% at a 95% level of confidence. Cows were selected by choosing every third cow in the shed or the yard. The cows observed for the assessment of AD were of indigenous Indian breeds (48.6%) which were Gir, Red Sindhi, Tharparkar, Kankrej, Sahiwal, Dangi, Deoni, Hariana, Nimari, Khillari, Nagauri, Rathi, and Pahari, cross breds with exotic cows (29.1%), cross breds between indigenous breeds (21.5%) and very few pure bred exotic breed (0.7%) of Jersey and Holstein Friesian type. Data collection included recording of direct observations of the cows and cow measurements (animal-based parameters), as well as the recording of resource-based parameters in the shelters and a structured interview of the shelter managers. All the recordings were performed by one single assessor (AS), who undertook a three-month training in scoring the cows for AD, BCS (body condition score), lameness, claw overgrowth, dirtiness, lesions on the limbs, joints and body, rumen fill, faecal consistency, and skin tenting time at the University of Queensland’s School of Veterinary Science. In order to validate the selected welfare measures, pilot trials were conducted in two shelters before the commencement of actual study (Tables S1 and S2).

### 2.1. Cow-Based Measures

A total of 1620 cows were assessed for the 27 animal-based parameters based on a literature search (mainly taken from the Welfare Quality multi-criteria model) [34,35], and our experience of welfare issues in shelters. Lactation status and age of the cows were ascertained from the physical examination and the interview of the shelter manager. The details of the scoring systems followed on the welfare assessment of individual cows in the study are listed in Appendix A Table A1.

The avoidance distance was assessed at the beginning of the shelter visit one hour after the morning feeding of the cows, as recommended in the Welfare Quality^®^ protocol [36]. A cow was approached immediately in front at a rate of 1 step/s, starting at 2 m from the manger. The distance between the assessor’s hand and the cow’s head was estimated at the moment the cow moved away and/or turned its head, in the following four categories: touched, and hand within 50 cm, 51–100 cm, and >100 cm. For each shelter, the median AD classification and % of cows which could be touched on the head were calculated. In shelters where cows were tethered, they were untied and moved outside the shelter to assess AD and lameness, and then retied for all remaining animal-based measures.

Body condition score (BCS) was determined using a 1–5 scale [37,38] and scored to quarter points. Each sampled cow’s demeanour was assessed during restraint on a dichotomised scale (docile or aggressive), which was derived from a five-point scale [39] for loosely restrained cattle in a particular area of the shelter shed.

### 2.2. Health Measures

Dirtiness of the hind limbs, udder, and flanks was classified by visual inspection of the cows from the left side, right side, and from behind, according to the method of Whay et al. [40]. The body hair loss score was assessed as per the method described by Whay et al. [40]. Tarsal lesions assessment included hair loss, ulcerations, and swellings, a modification of the methods of Wechsler et al. [41] and Whay et al. [40]. Carpal joint injuries were scored according to the method of Wechsler et al. [41]. Neck lesions were classified according to the method of Kielland et al. [42]. Respiratory problems were measured as the presence or absence of coughing in any of the 30 cows sampled in the sheds during the total examination period of the sampled cows in each shed. A cow expressing frequent coughs (more than five) during the 10–15 min examination time for assessment of the welfare parameters was considered to be having respiratory problems. Ocular lesions, nasal discharge, hampered respiration, diarrhoea, and vulvar discharge were assessed on a binary scale, i.e., present or absent in the sampled cows [43]. Rumen fill score (RFS) was visually scored according to the method of Zaaijer and Noordhuizen [44], standing behind the cow on the left side and observing the left paralumbar fossa between the last rib, the lumbar transverse processes, and the hip bone. The consistency of the faeces of the sampled cows was visually inspected and rated on a five-point scale formulated by Zaaijer and Noordhuizen [44]. Skin lesions or integument alterations on the body were recorded using the method of Leeb et al. [45]. Hair Coat condition was assessed as per a modified scale derived from Huxley and Whay, modifying their categorization from dull, thick or excessively hairy to dull and short, shiny and short, or dull and hairy [46]. Claw overgrowth was visually inspected on each sampled cow and scored according to the scale devised by Huxley and Whay [47]. Lameness was scored using a numerical rating scale for walking cows (1—not lame to 5—severely lame) followed by Flower and Weary [48] and Sprecher et al. [49]. Ectoparasitism was assessed by visual examination of each sampled cow as per the method described by Popescu et al. [50]. The protocols for teat and udder scoring (score 0–5) and skin tenting time (score 1–3) were designed by the authors, because of anticipated emaciation, teat and udder abnormalities, and advanced age would be more common in the shelters than in dairy cow farms, for which other scales are designed. Dehydration was assessed with skin turgor meaning the time a skin tent takes to return to its original position period [51]. The scoring pattern and scales for skin tenting time and teat and udder abnormalities are also described in Appendix A Table A1 for easy reference.

### 2.3. Shelter-Based Measures

The total number of sheds per shelter and the number of cows per shed in the shelter was assessed by visual inspection (the latter using a maximum of two sheds per shelter). The length, breadth, and height of the sheds were recorded using a laser distance meter (CP-3007 model, Ultrasonic distance meter 40KHz frequency, Chullora, New South Wales, Australia) and confirmed using a traditional measuring tape each time. From these measurements, the area of the shed and area per cow was calculated. The space allowance per cow in shelters having loose housing was calculated by dividing the floor area of the shed by the total number of cows within the shed. In shelters with stalls, the area/cow was calculated using the floor area of each stall housing a cow [52,53]. In tethered stalls, the area per cow was calculated by measuring the distance from the end of the rope at the point of attachment to a peg to the end of the hind limb of the cow at full extension. This length was used as a radius to calculate the maximum potential area of movement of the tethered cows in the sheds. The number of cows per shed was also counted during inspection of the sheds.

The gradient of the floors in the sheds and the yards were measured at three different places using vertical and horizontal measurements at each place using an inclinometer (Bosch Professional, 600MM, DNM60L Model, Clayton, Australia). The traction of the floors was determined as the coefficient of friction (CoF) (the force required to move an object over a floor divided by the weight of that object) [54,55]. This was estimated using a 1 kg/10 N spring balance attached by a hook to a cuboid wooden block (mass 156 g). The block was gently pulled across the floor at a speed of 0.17 m/s and the minimal frictional force (F) required to keep it moving was recorded [56].

The type of shed flooring (brick, stone, earthen, concrete, or other), presence of bedding in the sheds (present or absent, if present its thickness), type of bedding if present (hay, straw, rubber mats or other) and presence of yards were recorded during the inspection of the shelter facilities [52,57,58]. The cleanliness of the shelter premises was recorded by visually estimating the mean percentage of the floor that was covered by dung and urine in each shed, passage, and yard separately, as % of the area covered by dung in the shed lying areas and passages, urine in the shed lying areas and passages (present or absent), run-off in the shed (present or absent), and cleaning frequency of floors of the sheds [59].

## 3. Statistical Analysis

Descriptive, principal component analysis (PCA), Spearman’s rank correlation, and multivariate analyses were conducted using statistical software Minitab.17.1.0. Variables were tested for normality by the Anderson–Darling test [60]. We generated two models for the data analysis. In the first, cow specific risk factors for AD were examined by multivariate analysis of the animal-based measures. An ordinal logistic regression analysis was conducted using the four AD scores as outcome variable. Categorical parameters having more than three categories were treated as continuous variables. Observations within shelters were accounted for by including shelter as a clustering effect in the model.

In the second model, resource-based parameters were analyzed at the shelter level. Shelter level AD estimates were used as the outcome in analyzing the risk factors. A principal component analysis (PCA) was employed to reduce the number of variables and to minimize the multicollinearity. The resource-based variables dropped from the analysis were the % of dung lying in the shed passages, the thickness of shed bedding and % of urine in the shed passages. Univariate analysis was conducted to explore associations between the variables using Spearman’s rank correlation because the variables were not normally distributed as ascertained by the Anderson–Darling test. The multivariate analysis of the resource-based parameters with AD was done by a stepwise selection of terms in a general linear model (GLM) with α to enter at 0.15. The residuals were analyzed to explore the basic assumptions of logistic regression and model fit according to Dohoo et al. [61]. Levels of significance were set as *p* ≤ 0.05 for all the analyses. The residuals were normally distributed (*p* = 0.12) and were also inspected graphically. The r^2^ (adjusted) for this dataset was 45.9%.

## 4. Results

Descriptive statistics for cow-based and shelter-based parameters are shown in Table 1 and Table 2. None of the parameters were normally distributed.

### 4.1. Cow-Based Measures

In the AD test, one half of the cows (51.2%) allowed themselves to be touched and most of the other half (46.6%) had an avoidance distance up to 100 cm (scores 1 and 2) (Table 1). As a precondition in our study, the majority of the cows were non-lactating (87.9%). The physical and clinical examination revealed that the majority of cows (76.4%) were of a docile temperament. The median age of the cows was 11 years and median BCS 2.68. The majority of the cows (75.4%) were in the normal BCS category scores (between 2.25–3.75).

The dirtiness of the hind limbs (85.6%), udder (76%), and flanks (74.2%) was mostly in the mild and medium categories. There was no body hair loss in almost half of the cows (45%) and a mild hair loss in other one third (30.3%). Tarsal joint swellings (86.1%) and hair loss (76.7%) were predominantly in the mild to moderate category scores. The majority of cows (86.9%) either had no or mild tarsal joint ulcerations. More than half of the cows (54.9%) had mild to moderate carpal joint injuries.

The prevalence of neck lesions, ocular lesions, coughing, nasal discharge, hampered respiration, diarrhoea, and vulvar discharge was predominantly below 10% in the shelter cows. Body/skin lesions were absent in nearly half of the cows and the other half had mild to moderate lesion scores. Body coat condition was almost equally distributed between dull and short coats (47%) and shiny and short coats (52%). The rumen fill score, which is an assessment of the dry matter intake, ration composition, digestion and rate of passage of ingesta [44], was scored as 3 and 4 in most of the cows, which are usually the common scores for lactating and dry cows. Faecal consistency scores were 3 and 4 in the majority of the cows. 

Moderate to severe claw overgrowth was observed in 11.1% cows only. Clinical lameness (score 3 to 5) was present in only 4.26% cows. Teat and/or udder abnormalities were observed in very few cow (2.3%) cows. Skin tenting time representing dehydration, was normal (≤2 s) in 92.2% cows.

### 4.2. Shelter-Based Measures

The median number of cows per shed was 70 and the median area per cow was 2.73 m^2^ (Table 2). The average gradient of the flooring of lying areas and passages of the sheds was 1.46% and 2.36%, respectively. The CoF of shed flooring was 0.43. The median % dung in the lying areas and passages of the sheds was 15% and 10%, respectively.

Half of the shelters had concrete floors followed by earthen (24%), brick (22.2%), and stone (3.7%) floors. There was absence of bedding in majority of the shelters (96.3%) and the only two shelters which provided bedding used paddy straw of 0.05 cm thickness or less. In 87% of the shelters, yards were present for the cows to loaf out of the sheds. The median dry bulb temperature and humidity in the sheds was 25.5 °C and 34%, respectively. The median luminosity in the sheds was 582 Lux and the median noise level was 27.6 decibel.

### 4.3. Relationship between Cow-Based Measures and Avoidance Distance

The univariate analysis of the cow-based welfare measures at cow level using the Spearman’s rank correlation (Table 3) revealed significantly positive correlation between AD and BCS, dirty udder, dirty flanks, body hair loss, tarsal joint ulceration, carpal joint injuries, ocular lesions, nasal discharge, diarrhoea, lameness, lesions on the body, claw overgrowth, coat condition, ectoparasitism, skin tenting time, and age of the cows. There was a significantly negative correlation between AD and general demeanour, rumen fill score, and faecal consistency.

In multivariate analysis, the ordinal logistic regression was used to examine the relationship between the cow-based welfare measures as predictors and the AD as the ordinal response variable. The BCS, dirty hind limbs, dirty flanks, tarsal joint swelling, tarsal joint hair loss, tarsal joint ulceration, carpal joint injuries, hampered respiration, diarrhoea, rumen fill score, lesions on the body, and coat condition of the cows were significantly associated with AD as an ordinal outcome variable (Table 4). The odds of a greater AD was negatively associated with their BCS (OR = 0.57, CI = 0.46–0.71).

In relation to health measures, the odds of a greater AD was positively associated with dirty hind limbs of the cows, but negatively associated with dirty flanks. The odds of a greater AD were positively associated with tarsal joint swellings and tarsal joint hair loss, but negatively associated with tarsal joint ulceration, carpal joint injuries, lesions on the body, and coat condition of the cows. They were also negatively associated with the presence of hampered respiration and diarrhoea. The odds of a greater AD were positively associated with rumen fill score.

### 4.4. Relationship between Avoidance Distance and Shelter-Based Measures

The univariate analysis of the shelter-based measures by Spearman’s rank correlation (Table 5) found a significantly positive correlation (*p* < 0.05) between AD and cows/shed, luminosity in the sheds and noise levels in the sheds. There was a significant negative correlation (*p* < 0.05) between AD and shed area/cow and shed dry bulb temperature.

In the multivariate analysis model (Table 6; r^2^ adjusted = 45.9%; residuals normally distributed, *p* = 0.12), AD had a significant positive association with noise levels in the sheds and cleaning of the sheds. There was a significant negative association of the AD with % dung in the lying areas, dry bulb temperature, and humidity. The relationship was described by the equation:

Avoidance Distance = c + 3.87 (±0.506, *p* ≤ 0.001) − 0.008% dung in the shed lying area (±0.002, *p* = 0.004) + 0.008 shed noise level (±0.003, *p* = 0.02) − 0.04 shed dry bulb temperature (±0.011, *p* ≤ 0.001) − 0.02 shed humidity % (±0.004, *p* ≤ 0.001) + 0.21 cleaning of sheds (±0.084, *p* = 0.01) (1)

where c is the intercept, which was 4.10 for sheds that were cleaned and 3.66 for sheds which were not cleaned.

## 5. Discussion

Avoidance distance of cows towards an unfamiliar human has been validated as a stable behaviour indicator of human–animal relationship [62,63,64]. These studies measured AD at the feeding manger and validated the protocols to assess the human–animal relationship. This test of assessment of the human–animal relationship is easy to perform in an on-farm welfare assessment and has a high correlation with avoidance distance in a pen and moderate correlation with cows’ response to a human walking through a herd and touching standing or lying cows [65]. We replicated the welfare quality protocol quite closely—i.e., the avoidance distance of 30 cows in each shelter in the morning one hour after the feeding time [34,35]—and utilized the data generated to determine correlations between AD and other measures, and reported these, together with the proportions of cows with AD of zero and the median AD score. More than half of the cows in our study had an avoidance of zero (allowing touch), which is proportionately higher than the European dairy cattle herds, which had a wide range of 2–67% of cows [7,62]. Australian dairy herds have been measured with 30% with this score [19,25]. In terms of AD, the sheltering of cows may be more similar to the traditional management of dairy cows, which had frequent contact with handlers during feeding, watering, and cleaning [66], in contrast to the present day intensively managed factory farming of dairy cows. The results of our study indicate an overall good level of the human–animal relationship and reflect a high level of confidence for the cows in the presence of humans, as inferred by previous authors that have used the measure and discussed its relevance [67]. The cows which allowed touch by humans may be assumed to be the ones with very good human–animal relationships [65].

### 5.1. Relationship between Cow-Based Measures and AD

The human–animal relationship has been found to have some correlations with health in dairy cattle: positive interactions between the stockpersons and their cows can reduce somatic cell numbers in milk [40]. However, these relationships are generally unexplored. 

The significant negative association between AD and BCS in this study could be due to the low BCS cows being weak and energy deficient, body condition being a general indicator of health and nutrition of the cows [66,68]. Thus, they were not able to move away from an approaching stranger. Similar findings were reported in a French dairy herd where more cows with low BCS allowed being touched by the observer at the feeding rack [65]. Another explanation could be that since the cows were tested in the morning at the shelter feed bunks, in the low body condition cows a higher motivation could exist for feeding than escaping from the observer [65].

The significant association of dirty hind limbs with AD could be explained by cows with a higher AD moving away more from approaching strangers and get their limbs soiled due to the slurry present in the lying areas and passages of the sheds. Dirtiness scores of the hind limbs have been associated with contamination of the floor surface of the dairy stalls [69]. Conversely, the negative association of dirty flanks with AD could be attributed to cows suffering from diarrhoea being reluctant to move. Diarrhoea renders cows weak due to loss of energy, electrolytes, and subsequent by dehydration. The loose faeces which soils the tail is often transferred to the flanks, rendering the flanks dirty [70]. The relationship is further supported by the negative association of AD with cows having diarrhoea. We tested the correlation between AD, diarrhoea dirty flanks, and lameness using the Spearman’s rank correlation and found significant (*p* ≤ 0.001) relationships between AD and diarrhoea (r_s_ = 0.158, *p* ≤ 0.001), dirty flanks (r_s_ = 0.216, *p* ≤ 0.001), and lameness (r_s_ = 0.119, *p* ≤ 0.001). There are several possible explanations for these associations. Lame cows may spend more time lying down due to the pain of lameness, with greater chance that they will lie on dung [71]; lame cows pass urine and dung while lying as they find difficulty in standing [72]; during standing lame cows also might have difficulty to adopt a normal urination and defecation posture, making the lying area dirty and wet, leading to dirty hind limbs [73]. There is also likely to be a relationship between stockperson’s attitude towards animals and their health by virtue of the former’s approach to the maintenance of cleanliness of farm premises [74]. Intervention studies have shown that training of the stockpersons to improve their attitudes towards dairy cows reduced aversive handling of these animals, leading to reduced stress levels and improved productivity [75].

The significant positive association of AD with tarsal joint hair loss and swelling could be attributed to the higher AD in nervous cows which might sustain tarsal joint hair loss and swelling due to fear of an approaching human. Most of the shelters had no bedding and nervous cows get injured as they suddenly get up when threatened or collisions with shelter furniture. Tarsal lesions have been associated with abrasive surfaces of lying areas and inadequate design of the facilities [76,77,78]. The human–animal relationship has been identified as a possible factor affecting tarsal lesions in dairy cows because a negative human–animal relationship might affect the lying comfort of the cows as sudden rising movements out of fear of stockperson can lead to tarsal joint injuries [58].

The significant negatively association of AD with tarsal joint ulceration and carpal joint injuries could be explained by the reluctance of the cows to move away or move away slowly from the approaching human, due to the pain associated with these lesions. We found significant relationships in the univariate analysis using the Spearman’s rank correlation between AD and lameness (r_s_ = 0.119, *p* ≤ 0.001), tarsal joint ulceration (r_s_ = 0.154, *p* < 0.001), and carpal joint injuries (r_s_ = 0.232, *p* < 0.001). Lameness was not found to be a confounding factor to AD assessment in a study in Austrian dairy herds, but the researchers advocated further investigations of the relationship [29].

The significant negative association of AD with cows having hampered respiration in our study could also be due to the inability of the cows to move away from the approaching stranger, as a result of weakness or poor health. Visual examination of such signs is a significant aspect of health evaluation in cows.

The positive association of AD with rumen fill score could be due to the fact that cows consuming adequate feed were in a position to avoid the approaching experimenter. The rumen fill indirectly reflects adequate energy and alertness of the cows to avoid and move away from a stranger. The rumen fill score represents the amount of dry matter and fluid in the rumen [44]. It is related to dry matter intake, feed formulation, digestibility, and the rate of passage of ingested food through the alimentary tract [79,80]. This score has also been used to identify diseased cows, with a low score indicating poor condition. We cautiously interpret this association because the dry matter intake in cows varies over the day, which alters the rumen fill score [81]. However, we also observed a positive correlation between rumen fill score and BCS (Spearman’s rank correlation coefficient, r_s_ = 0.132, *p* ≤ 0.001). Our study is different from the previous studies on the association of rumen fill scores and health [44,82,83], as these focused on healthy lactating dairy cows whereas most of our cows were non-lactating and sustained on dry fodder only.

The significant negative association of AD with lesions on the body and coat condition of the cows could be explained by the poor health condition of the cows exhibited by these conditions. Research has demonstrated a strong relationship between chronic pain and generalized anxiety in humans leading to distress [84]. Furthermore, a biopsychosocial model of experiences of chronic pain has suggested that there is an interaction of physical trauma, psychology, and environmental factors [85]. A poor hair coat condition is a common clinical sign of chronic ill health status of the cows, which might due to feeding poor quality fodder, the lack of access to a balanced diet, inadequate fodder, or parasitism [46,86,87,88]. The poor health of the cows demonstrated by these alterations on the body and coat could have affected their strength to move away from the approaching experimenter due to chronic pain and generalized distress.

### 5.2. Relationship between Shelter-Based Resource Measures and AD

The negative relationship between AD and the % of dung in the lying area in the shelter sheds may reflect greater ease of movement of the cows away from the approaching person when the sheds were clean. The presence of dung mixed with urine to form a slurry affects the locomotion of the cows as it reduces the coefficient of friction of the flooring [88]. This reduction in the floor friction leads to slipping and cows adopt an unnaturally stiff gait [89,90].

A weak positive association of AD with noise levels in the shelters could be due to the cows’ getting alarmed and stressed by the noise. Cattle tend to get disturbed at noise levels above 90–100 dB [91] and dairy cows are more sensitive to noise than beef breeds [92]. The median noise levels in the shelters (27.6 dB) in our study was far below the threshold limits of getting alarmed and probably reflects the fact that most shelters were situated in quiet locations. Nevertheless, the noise had an effect on the AD, despite the observer remaining silent while approaching the cows to avoid affecting the sensitivity and temperament of the animal [92]. The weak association could be due to general agitation of the cows in noisier environments and the presence of novel sounds in the shelters [93], which affected the cows but was not detected at the time of our measurements, such as the noise of the shelter machinery and sounding of horns in shelters located near busy highways. Noise therefore clearly impacts on the behaviour of cows and this result suggests that it can adversely affect its welfare, hence this environmental parameter should be considered during designing and construction of cow housing [94].

The highly significant negative relationship between AD and dry bulb temperature and humidity levels in the shelters can be attributed to impact of the microclimate on the behaviour of cows [95,96,97]. The median dry bulb temperatures of 29.5% and 34% humidity levels found in the cow shelters depict moderate levels of heat stress. Heat stress may make cattle focus on coping with the high temperature rather than the threat posed by the person, which could be the reason for this relationship in our study.

There are differences between farms and animals in the extent to which animals are fearful of people. There are breed and individual differences in the degree of fearfulness in animals but much of the fear of humans is due to the way animals are handled [98]. We did not take into consideration the breed differences in cows in our study because the cows were predominantly of the local indigenous type. There is a linear relationship between stockpersons’ attitude, belief, and behaviour while handling animals and its effect on the animals [8]. Most of the pioneering correlational studies in this subject area describe the relationship between the manner of animal handling affecting the productivity and welfare, through fearfulness in animals [75,99,100,101].

The major limitation of the study was that, being a cross-sectional study, there was no confirmation of evidence of causation despite the correlations observed between AD and other welfare parameters. Nonetheless, our large sample size can provide reliability to the results. There is a risk that we did not measure all the important factors involved in cow health or shelter resources, leading to spurious correlations being observed. The inherent limitation of a cross-sectional study is its difficulty to identify the causal relationship between the variables. Some factors were difficult to identify, for example AD in cows could be influenced by the typical genotype of the cows, but we had insufficient clear evidence of distinct genotypic factors that we could include in the model. Some of the relationships could be influenced by associations between negative attitudes of stockpersons’ towards cows, which were correlated with careless attitudes towards other tasks of stockpersonship such as maintenance of cleanliness and good feeding practices. The multiple regression used in our study helps to identify the most important relationships but still the understanding of causal relationship requires intervention studies.

## 6. Conclusions

Relationships were observed between AD and other animal and resource-based welfare indicators in the cow shelters. The measurement of AD at the feed bunk appears to be a promising test for the assessment of human–animal relationship in the cow shelters but recording of cow health parameters and some resource-based parameters may help to explain variation between cows. Our results show that AD is dependent on various health and welfare parameters, which makes it relative to the state of the animal. We suggest a cautious interpretation as AD in circumstances in which health variables described in this study are influencing AD, as well as it being a reflection of stockpersonship. Thus, although previous studies have reported it to be a highly repeatable test [98], further refinement could improve its usefulness. The results of this study also suggest that the human–animal relationship in most cow shelters is cordial and in line with the animal welfare principles, because one half of the cows allowed touch by the assessor. Welfare assessment protocols for shelters could usefully include this measure. Further studies on the repeatability and validity of AD in cow shelters are needed, and this study can be regarded as a preliminary investigation into the human–animal bond in the cow shelters at a particular point of time. The low AD values observed in this study suggest that the positive behaviour of the handlers towards cows in the shelters have produced a good human–animal relationship, which helps in guaranteeing good cow welfare in the shelters.

## Figures and Tables

**Table 1:** Distribution of different cow-based welfare parameters in 54 cow shelters (*n* = 1620).

Parameter	% Score and Number					
	0	1	2	3	4	5
Avoidance distance score (Scale 0–3)	51.2 (830)	31.4 (508)	15.4 (249)	2.0 (33)	-	-
Lactation (0: non-lactating; 1: lactating)	88.0 (1425)	12.0 (195)	-	-	-	-
BCS	≤1.25 (emaciated) 0.1 (2)	1.5–2 (thin) 22.9 (371)	2.25–3.75 (normal) 75.5 (1233)	4 or more (obese) 1.5 (24)	-	-
General demeanour (0: docile; 1: aggressive)	76.4 (1238)	23.4 (382)	-	-	-	-
Dirty hind limbs score (Scale 0–3)	2.4 (38)	42.6 (690)	43.0 (697)	12.0 (195)	-	-
Dirty udder score (Scale 0–3)	17.5 (283)	44.6 (722)	31.4 (509)	6.5 (106)	-	-
Dirty flanks score (Scale 0–3)	19.6 (316)	42.2 (684)	32.0 (519)	6.2 (101)	-	-
Body hair loss score (Scale 0–3)	45.0 (728)	30.3 (492)	23.0 (373)	1.7 (29)	-	-
Tarsal joint swelling score (Scale 0–3)	11.8 (191)	22.4 (362)	63.7 (1032)	2.1 (35)	-	-
Tarsal joint hair loss score (Scale 0–3)	23.0 (372)	49.4 (800)	27.3 (443)	0.3 (5)	-	-
Tarsal joint ulceration score (Scale 0–3)	53.6 (869)	33.3 (539)	13.0 (210)	0.1 (2)	-	-
Carpal joint injuries score (Scale 0–3)	44.8 (726)	31.9 (516)	23.0 (373)	0.3 (5)	-	-
Neck lesions score (Scale 1–4)	-	5.4 (1546)	3.8 (62)	0.4 (6)	0.4 (6)	
Ocular lesions score (Scale 0–1)	91 (1474)	9.0 (146)	-	-	-	-
Lesions on the body score (Scale 0–3)	45.3 (734)	32.3 (524)	20.5 (332)	1.9 (30)	-	-
Body coat condition score (Scale 1–3)	-	47.1 (764)	52.0 (843)	0.8 (13)	-	-
Nasal discharge score (Scale 0–1)	90.7 (1470)	9.3 (150)	-	-	-	-
Diarrhoea score (Scale 0–1)	95.7 (1551)	4.3 (69)	-	-	-	-
Faecal consistency score (Scale 1–5)	-	0.3 (5)	4.9 (79)	35.1 (569)	58.3 (944)	1.4 (23)
Rumen Fill Score (Scale 1–5)	-	0.1 (2)	3.7 (60)	36.8 (594)	58.7 (952)	0.7 (12)
Lameness score (Scale 1–5)	-	84.7 (1373)	11.0 (178)	3.2 (53)	1.0 (15)	0.06 (1)
Claw overgrowth score (Scale 0–3)	52.5 (850)	36.4 (589)	9.6 (156)	1.5 (25)	-	-
Teat score (Scale 0–5)	14.5 (235)	83.2 (1348)	0.4 (6)	0.4 (7)	0.0 (0)	1.5 (24)
Ectoparasitism score (Scale 0–4)	0.4 (6)	53.1 (861)	34.5 (559)	11.8 (191)	0.2 (3)	-
Skin tenting time score (Scale 0–4)	92.2 (1494)	5.3 (86)	2.1 (35)	0.3 (5)	-	-

**Table 2:** Descriptive statistics of shelter-based resource measures (*n* = 54).

Parameter	Median	First Quartile (Q_1_)	Third Quartile (Q_3_)	Interquartile Range (IQR)
Cows/shed	70	47.8	137.3	89.5
Area/cow (m^2^)	2.73	1.56	3.62	2.06
Gradient of shed lying area flooring (%)	1.46	0.96	2.20	1.23
Gradient of shed passage flooring (%)	2.36	1.27	3.52	2.24
CoF of shed flooring	0.43	0.27	0.65	0.37
% dung in lying areas of shed	15	5	40	35
% dung in passages of shed	10	5	42.5	37.5
Dry bulb temperature of the shed (°C)	29.5	27.2	32.8	5.6
Shed humidity (%)	34	24.7	45.2	20.5
Shed luminosity level (Lux)	582	89	1036	946
Shed noise levels (Decibel)	27.6	21.3	37.1	15.8

**Table 3:** Spearman’s rank correlations between avoidance distance scores for each cow (*n* = 1620) and cow-based welfare parameters.

Parameter	Variables	Correlation Coefficient (r_s_)	*p*
Avoidance Distance (Score 1–4) 0—touched 1—50 cm to >0 cm 2—100 cm to >50 cm 3—>100 cm	Carpal joint injuries	0.232	≤0.001
	Dirty flanks	0.216	≤0.001
	Dirty udder	0.186	≤0.001
	Claw overgrowth	0.173	≤0.001
	Diarrhoea	0.158	≤0.001
	Lesions on the body	0.155	≤0.001
	Tarsal joint ulceration	0.154	≤0.001
	Skin tenting time	0.138	≤0.001
	Lameness	0.119	≤0.001
	BCS	0.093	≤0.001
	Body hair loss	0.090	≤0.001
	Age of the cows	0.082	0.001
	Ectoparasitism	0.063	0.01
	Ocular lesions	0.055	0.02
	Nasal discharge	0.056	0.02
	Coat condition	0.056	0.02
	Rumen Fill Score	−0.279	≤0.001
	General demeanour	−0.069	0.005
	Faecal consistency	−0.071	0.004

**Table 4:** Association of avoidance distance of shelter cows (*n* = 1620) with animal-based parameters using ordinal logistic regression.

Predictor	Mean	Coefficient	SE Coefficient	*p*	Odds Ratio	95% CI
Dirty hind limbs		0.68	0.114	0.000	1.98	1.58–2.48
Rumen fill score		0.58	0.093	0.000	1.79	1.49–2.15
Tarsal joint swelling		0.24	0.080	0.002	1.28	0.09–1.50
Tarsal joint hair loss		0.23	0.095	0.012	1.27	1.05–1.53
Lesions on the body		−0.22	0.018	0.006	0.80	0.68–0.94
Tarsal joint ulceration		−0.27	0.091	0.002	0.76	0.63–0.91
Carpal joint injuries		−0.33	0.070	0.000	0.72	0.62–0.82
Coat condition		−0.39	0.129	0.002	0.67	0.52–0.87
BCS		−0.56	0.109	0.000	0.57	0.46–0.71
Dirty flanks		−0.58	0.150	0.000	0.56	0.42–0.75
Diarrhoea						
Reference 0	1.65					
Reference 1	2.34	−0.72	0.280	0.010	0.48	0.28–0.84
Hampered respiration						
Reference 0	1.67					
Reference 1	2.57	−1.71	0.736	0.020	0.18	0.04–0.76

**Table 5:** Spearman’s rank correlations between mean shelter (*n* = 54) avoidance distance scores of the selected cows and shelter-based welfare parameters.

Parameter	Variables	Correlation Coefficient (r_s_)	*p*
Avoidance distance (Score 1–4) 0—touched 1—50 cm to >0 cm 2—100 cm to >50 cm 3—>100 cm	Cows/shed	0.337	0.01
	Shed average luminosity	0.293	0.03
	Shed noise levels	0.278	0.04
	Shed area/cow	−0.308	0.02
	Shed dry bulb temperature	−0.416	0.002

**Table 6:** Regression analysis of shelter-based measures significantly related (*p* < 0.05) to avoidance distance score.

Term/Parameter	Coefficient	SE Coefficient	*p*
Constant	3.87	0.506	≤0.001
Shed clean at the time of measurement	0.21	0.084	0.01
Noise levels in the shed (decibels)	0.008	0.003	0.02
Shed humidity (%)	−0.02	0.004	≤0.001
Dry bulb temperature in the sheds (°C)	−0.04	0.011	≤0.001
% dung in the lying area of the shed	−0.008	0.002	0.004

## Supplementary Materials

The following are available online at https://www.mdpi.com/2076-2615/9/7/396/s1, Table S1: Cow level data for Avoidance Distance in shelter cows, Table S2: Shelter level data for Avoidance Distance in Shelter cows.

## Appendix A

**Table A1:** Description of various cow-based welfare measures.

Parameter (Reference)	Description	Scales and Scores
Avoidance distance (AD) [36]	Cows that were standing at the feeding manger were approached at the front at a rate of one step per second, starting at 2 m from the manger. The distance between the assessor’s hand and the cow’s head was estimated at the moment the cow moved away and turned its head	0—touched; 1—0 to 50 cm; 2—51 cm to 100 cm; 3—>100 cm.
Lactation		0—non-lactating; 1—lactating.
General demeanor [39]	Visual examination	0—docile; 1—aggressive.
Body condition score (BCS) [37,38]	A cow with a score of ≤1.25 was considered emaciated, 1.5–2 thin, 2.25–3.75 normal and 4 or more obese; Visual examination	1 to 5 with increments of 0.25.
Dirtiness of the hind limbs, udder, and flanks [40]	By visual inspection of the cows from both sides (left and right) and from behind	1—no dirtiness; 2—mildly dirty (small soiled areas of dirtiness with no thick scabs); 3—medium dirtiness (large soiled areas but with <1 cm thick scabs of dung); 4—severely dirty (large soiled areas with >1 cm thick dung scabs).
Body hair loss [40]	Visual inspection	0—absence of hair loss; 1—mild hair loss; 2—medium hair loss; 3—severe hair loss.
Tarsal joint swellings [40,41]	Visual examination	1—mild swollen joint; 2—medium swollen joint; 3—severely swollen joint.
Tarsal joint hair loss and ulceration [40,41]	Visual examination	0—no hair loss or ulceration; 1—mild hair loss or ulceration <2 cm^2^; 2—medium hair loss or ulceration (approx. 2.5 cm^2^); 3—severe hair loss or ulceration >2.5 cm^2^.
Carpal joint injuries [41]	Visual examination	0—no skin changes; 1—hairless; 2—swollen; 3—wound(s).
Neck lesions [42]	Visual examination	1—no observable skin changes; 2—hair loss; 3—swollen; 4—closed wounds (haematomas or closed abscesses); 5—open wounds.
Ocular lesions [43]	Visual examination	0—absent; 1—present.
Coughing	Examination of the sampled cows	0—absent; 1—present.
Nasal discharge [43]	Visual examination	0—absent; 1—present.
Hampered respiration [43]	Visual examination	0—absent; 1—present.
Diarrhoea [43]	Visual examination	0—absent; 1—present.
Vulvar discharge [43]	Visual examination	0—absent; 1—present.
Rumen fill score [44]	Visually by standing behind the cow on the left side and observing the left para lumbar fossa between the last rib, the lumbar transverse processes and the hip bone	1—the para lumbar fossa is empty, presenting a rectangular cavity that is more than a hand’s width behind the last rib and a hand’s width under the lumbar transversal processes; 2—the para lumbar fossa forms a triangular cavity with a width about the size of a hand behind the last rib, but less than this under the lumbar transverse processes; 3—the para lumbar fossa forms a cavity less than a hand’s width behind the last rib and about a hand’s width vertically downwards from the lumbar transverse processes and then bulges out; 4—the para lumbar fossa skin covers the area behind the last rib and arches immediately outside below the lumbar transverse processes due to a bloated rumen; 5—the rumen is distended and almost fills up the para lumbar fossa; the last rib and the lumbar transverse processes are not visible.
Faecal consistency [44]	Visual inspection	1—thin and watery and not truly recognisable as faeces; 2—thin custard-like consistency, structurally recognisable as faeces, splashing out wide upon falling on the floor; 3—thick custard-like consistency, making a plopping sound while falling on the floor and a well-circumscribed pad which spreads out and is about 2 cm thick; 4—stiff with a heavy plopping sound while falling on the floor and a proper circumscribed pad with visible rings and minimal spreading out; 5—hard faecal balls like horse faeces.
Skin lesions/Integument alterations [45]	Visual examination	0—normal (no apparent lesions);1—mild hair loss (<2 cm^2^); 2—moderate (>2 cm^2^ hair loss and inflamed skin); 3—severe (a large >4 cm^2^ area of hair loss with extensive skin inflammation and breakage).
Body coat condition [46]	Visual examination	1—dull and short; 2—shiny and short; 3—dull and hairy.
Claw overgrowth [47]	Visual examination	0—normal claws; 1—mild claw overgrowth; 2—moderate claw overgrowth; 3—severe claw overgrowth.
Lameness score [48,49]	1 to 5 scale Visual examination	1—not lame (smooth and fluid movement); 2—mildly lame but not observable easily (an imperfect gait but able to freely move with a mildly arched back); 3—moderately lame (able to move but not freely, with an arched back) 4—lame, with the inability to move freely with and asymmetrical gait and abnormal head movement; 5—severely lame (severely restricted in movement, requiring considerable encouragement to move, and a severely arched back).
Ectoparasitism [50]	Visual examination	1—absence of ectoparasites; 2—mild infestation—no lesions (not easily visible by naked eye but on tactile perception in the neck region; 3—moderate-mild infestation visually observable ectoparasites or immature forms or eggs in the neck, groin, peri rectal, tail root and switch regions; 4—severe-visually observation of mature ectoparasites all over the body especially regions mentioned in score 3.
Teat and/or udder condition	Visual inspection	0—lactating udder and teats; 1—non-lactating udder and teats; 2—teat cracks; 3—warts on teats and udder; 4—acute lesions on the teats and udder; 5—chronic lesions on teats and udder.
Skin tenting time [51]	Visual examination by skin pinch of the cervical region of the neck	1—≤2 s; 2—>2 s; 3—≥6 s.
